# Convergent Validity of Three Measures of Reflective Function: Parent Development Interview, Parental Reflective Function Questionnaire, and Reflective Function Questionnaire

**DOI:** 10.3389/fpsyg.2020.574719

**Published:** 2020-12-16

**Authors:** Lubna Anis, Grace Perez, Karen M. Benzies, Carol Ewashen, Martha Hart, Nicole Letourneau

**Affiliations:** ^1^Faculty of Nursing, Cumming School of Medicine (Pediatrics), University of Calgary, Calgary, AB, Canada; ^2^Faculty of Nursing, University of Calgary, Calgary, AB, Canada; ^3^Faculty of Nursing, Cumming School of Medicine (Pediatrics and Psychiatry), University of Calgary, Calgary, AB, Canada

**Keywords:** reflective function, measurement, reflective function scale, reflective function questionnaire, parental reflective function questionnaire, convergent validity, APrON study

## Abstract

Reflective function (RF) is defined as an individual’s ability to understand human behavior in terms of underlying mental states including thoughts, feelings, desires, beliefs, and intentions. More specifically, the capacity of parents to keep their child’s mental states in mind is referred to as parental RF. RF has been linked to adult mental health and parental RF to children’s mental health and development. The gold standard measure of RF is the interview-based Reflective Functioning Scale (RFS) applied to the Parent Development Interview (PDI) or Adult Attachment Interview (AAI), which while well validated, is time-and labor-intensive to administer. Given the increasing need for reliable, valid, and rapid RF assessment in wide-ranging settings, two alternative measures were considered including the Reflective Function Questionnaire (RFQ) and Parental Reflective Function Questionnaire (PRFQ). We determined the convergent validity of these measures in comparison with the PDI-rated RFS. A sample of mothers and fathers (*n* = 150) was drawn from a sub-study of the ongoing Alberta Pregnancy Outcomes and Nutrition (APrON) longitudinal cohort when their children were 42–60 months of age. Pearson correlations and multiple linear regression was conducted, followed by splitting the sample to compute Cohen’s kappas measures of agreement. Two subscales of the PRFQ correlated significantly (*p* < 0.05) with the gold standard PDI-rated RFS, providing evidence for convergent validity. As a brief multidimensional measure of parental RF, the PRFQ offers an alternative for measurement of RF in large-scale studies of parental development and child health.

## Introduction

In recent years, the concept of Reflective Function (RF) has gained attention in research and clinical practice in both health and social sciences (Fonagy et al., 2002; Ordway et al., 2014; Antonsen et al., 2016). RF is defined as an individual’s capacity to perceive human behavior considering underlying mental states including thoughts, feelings, desires, and intentions (Fonagy and Target, 1997; Midgely and Vrouva, 2013). Early work describing RF referred to adults’ ability to envision mental states in themselves and in others (Fonagy et al., 1991a, b). Subsequently, researchers elaborated on this work to particularly address the capacity of parents to understand their children’s mental states to regulate their behavior, referred to as parental RF (Fonagy and Target, 1998; Slade, 2005). The capacity for being reflective is thought to be fundamental to humans’ ability to navigate the social world (Bateman and Fonagy, 2012). Impairments in RF have been associated with poor mental health such as depression (Lemma et al., 2011; Luyten et al., 2012a; Ekeblad et al., 2016) and borderline personality disorder (Fonagy and Bateman, 2007, 2008; Fischer-Kern et al., 2015). Reduced parental RF has also been associated with poor child development (Ensink et al., 2017; Borelli et al., 2019; Morosan et al., 2020).

Many measures are currently available to assess RF (Luyten et al., 2012b; Schiborr et al., 2013); however, the only well-validated gold standard measure that directly assesses RF is the interview-based Reflective Functioning Scale (RFS; Fonagy et al., 1998), typically applied to the Adult Attachment Interview (AAI; George et al., 1996) or the Parent Development Interview (PDI; Slade et al., 2004; Sleed et al., 2020). This interview-based measure of RF yields clinically rich data from which a RF score may be determined (Bouchard et al., 2008), but interviews often take 1–3 h to administer, followed by 2–4 of coding by highly trained professionals (De Roo et al., 2019). Therefore, there is a need for more time-saving and cost-efficient tools suitable for use in large-scale studies or in community settings. Two such measures include the Reflective Function Questionnaire (RFQ; Fonagy et al., 2016) and Parental Reflective Function Questionnaire (PRFQ; Luyten et al., 2017a, b), which exhibit good construct validity, internal consistency, and reliability (Rutherford et al., 2015; Fonagy et al., 2016; Luyten et al., 2017a). The RFQ has been compared with the gold standard RFS applied to the PDI in only one study with mixed results (Handeland et al., 2019). However, the PRFQ has never been compared with the gold standard RFS to our knowledge. Thus, the purpose of the current investigation was to determine the convergent validity, that is, the degree to which the RFQ and PRFQ correlate with the gold standard RFS, applied to the PDI (Karp et al., 2018).

### Origins of RF

Fonagy et al. (1991a, b) from the University College London were the first to introduce the construct of RF, proposing that RF is the operationalization of mentalization theory. Mentalization refers to the capacity to evoke, understand, and reflect on one’s own and others’ state(s) of mind while having insight into what one is (and others are) feeling and why. Fonagy et al. (1991a) proposed that the ability to mentalize develops as a function of the parent’s accustomed reading and regulating of their child’s internal state from the child’s perspective. This capacity enables the parent to understand their child as separate from the parent with mental states that are distinct from those of the parent and others (Winnicott, 1962; Fonagy and Target, 1998). Building on Fonagy’s work, Slade (2005) formally introduced the concept of *parental* RF to include parental ability to mentalize about themselves and their child. Parental RF is distinct from general RF as the concept of parental RF is more parent–child relationship-specific (Fonagy and Target, 1998; Slade, 2005). Although general RF and parental RF may be expected to be correlated, these two capacities may not be similar (Fonagy et al., 2016) and a correlation of *r* = 0.50 has been reported between general RF when scored from the AAI (George et al., 1996) and parental RF as scored on the PDI (Slade et al., 2004; Steele et al., 2008; Sleed et al., 2020).

There is growing consensus that RF is a developmental attainment that is sensitive to the effect of early interactions with primary caregivers (Fonagy and Target, 1997; Fonagy et al., 2003; Steele et al., 2008). Mothers’ disposition to reflect on and elaborate on about mental states is linked with their children’s social-emotional development (Laible, 2004; Garner et al., 2008; Meins et al., 2013). The importance of maternal RF for children’s developmental outcomes can be attributed to its main role in the transmission of attachment security from the mother to the child (Slade et al., 2005; Steele et al., 2008). Fonagy et al. (2002) proposed a developmental model of RF, suggesting that awareness of mental states develops in the context of early attachment relationships, in which children learn to identify and reflect their own affects through observing the parent’s responsiveness in their subjective experience. Also, parental ability to imagine the subjective experience of their developing children is thought to expedite the development of children’s self-regulation, along with representation of and communication about affects (Fonagy et al., 2002). Empirically, parental RF is associated with infant attachment (Stacks et al., 2014; Ensink et al., 2016) along with child and adolescent RF (Ensink et al., 2016; Duval et al., 2018). Furthermore, parental RF focused intervention demonstrated improvement in maternal RF in a sample of incarcerated women with custody retention issues (Baradon et al., 2008; Sleed et al., 2013).

### Measures of RF

The RFS is the primary psychometric tool used to measure capacity for RF (Fonagy et al., 1998). An elaborate coding procedure is required based on the narratives from AAI (George et al., 1996), and PDI (Slade et al., 2004; Sleed et al., 2020). Trained coders provide a global score on an 11-point scale that ranges from antireflective to exceptionally reflective. The RFS exhibits good inter-rater reliability after training (Fonagy et al., 1998), with Pearson correlations ranging between 0.86 and 0.91 (Bouchard et al., 2008), as well as an intra-class correlation coefficient of 0.86 (Levy et al., 2006). The RFS has been operationalized into two overlapping, yet different, measurement constructs, i.e., adult/self and parental RF. Whereas adult/self RF is principally rated from the AAI and spotlights adults’ ability to reflect on their childhood experiences with parental figures in mentalizing terms, parental RF has a more specific focus on parental capacity of the self/adult to mentalize about and reflect on their relationship with their children (principally rated from interviews on parenthood) (Slade et al., 2003; Slade, 2005).

The requirement for time-consuming interviews, specialist training, and costly human resources hamper research efforts and limit the RFS’s utilization outside of research settings, which is unfortunate, considering the affluent clinical potential implicit in the assessment of RF. The RFS has also been criticized for generating only global scores for *overall*, adult/parent and *child* RF, which may fail to address the complexity of the mentalizing process (Taubner et al., 2013). In seeking other options, Schiborr et al. (2013) identified different measurement approaches to assessing RF-related concepts. These approaches include assessments of insightfulness (Bretherton et al., 1989; Oppenheim and Koren-Karie, 2009), meta-emotional representation (Gottman et al., 1996), maternal mind-mindedness (the propensity to attribute meaning to the child) (Meins, 1998), the use of mental-state terms (Ruffman et al., 2002), mental-state language (Schechter et al., 2006), and mental-state references (Slaughter et al., 2008). Again, their time- and cost-intensive nature and less direct focus on RF hampers their use in large-scale studies and routine clinical applications (Fonagy et al., 2016; Handeland et al., 2019).

There is a need for a short, self-report questionnaire to measure reflective function as an aspect of parenting for use with mothers and fathers. This tool would be a valuable screening measure in the context of time- and resource-limited child health services, and for population-based research. Attempts to develop such measures include the RFQ (Luyten et al., 2012a) and the PRFQ (Fonagy et al., 2016). The RFQ exhibits good internal consistency reliability (α = 0.70 or greater). Convergent validity has been established between the two subscales of RFQ (RFQ Certainty and RFQ Uncertainty subscales) and allied concepts of alexithymia [with RFQ uncertainty, Spearman’s ρ(199) = 0.66, with RFQ Certainty, Spearman’s ρ(199) = −0.46, both *ps* < 0.001], empathy [with RFQ Uncertainty, Spearman’s ρ(209) = −0.37, and with RFQ Certainty, Spearman’s ρ(209) = 0.28], and mindfulness [RFQ Uncertainty, Spearman’s ρ(204) = −0.56, and with RFQ Certainty (Spearman’s ρ(204) = 0.39, both *p*s < 0.001)] (Fonagy et al., 2016). The test–retest reliability and internal consistency of the subscales were found to be satisfactory to excellent; the subscales were unrelated to demographic characteristics (Fonagy et al., 2016). Also, the PRFQ demonstrates good internal consistency for all subscales; Pre-mentalizing (α = 0.70), Interest and Curiosity (α = 0.74), and Certainty in Mental States (α = 0.82) (Luyten et al., 2017b).

To date, the validity and reliability of the RFQ and the PRFQ have been determined mostly in clinical, but not normative or non-clinical samples (Luyten et al., 2012a; Badoud et al., 2015; Fonagy et al., 2016; Morandotti et al., 2018). Moreover, the distinction between mothers’ and fathers’ capacity for RF have been examined predominately in clinical samples (Fonagy et al., 1991b; Cooke et al., 2017), with fathers’ RF given less attention. To develop a better understanding of parental RF of mothers and fathers, further research with a community sample is needed. The purpose of the current study is to assess the psychometric properties of the RFQ (Luyten et al., 2012b) and PRFQ (Fonagy et al., 2016) by determining the extent to which they correlate with the gold standard RFS applied to the PDI to establish convergent validity. Given the emphasis of both the PDI and PRFQ on parenting, we hypothesized that the PDI-rated RFS scores would correlate more strongly with the PRFQ than the RFQ.

## Materials and Methods

Mothers and fathers took part in a follow-up sub-study of their 3.5–5 years old children, enrolled in the Alberta Pregnancy Outcomes and Nutrition (APrON) longitudinal cohort study (Kaplan et al., 2014). The follow-up focused on parental RF, parent–child interaction quality, attachment, and child development in preschoolers and data were collected at clinic visits at the regional child development center by trained research assistants. Parents took part in a process of informed consent at enrollment in both the APrON study and follow-up sub-study. Honoraria were provided to compensate families for the burden of participating in the sub-study.

### Sample and Recruitment

Between 2011 and 2012, mothers were enrolled via maternity, ultrasound, and obstetrics clinics and media advertisements (Manca et al., 2013; Kaplan et al., 2014). Pregnant women were enrolled after 27 weeks gestation; they were over 16 years of age and English speakers. Mothers who were known or reported illicit drug users were ineligible. At enrollment, fathers were defined as males who self-reported to be the child’s father. At the 3.5–5 years follow-up clinic visit, mothers and fathers were recruited and enrolled in the present sub-study based on the following criteria: (a) child was in the target age-range of 60 months of age or less, i.e., preschooler; (b) family resided in the city where research was being conducted; (c) mother and father were in co-parenting relationship. To elaborate, participating fathers were expected to report being in a co-parenting relationship with the child’s mother, which meant engaged in day-to-day decision making about the child, but not necessarily co-habiting. Parental marital status was recorded. Both parents signed consents to participate in the follow-up. Families with a child who had a genetic disorder characterized by an intellectual or motor disability were excluded from this follow-up. Families were recruited until complete data were obtained on 75 mother–father pairs.

### Overview of Study Variables

At the 3.5–5 years visit, to characterize the sample, we collected data on parents’ demographic and descriptive characteristics (e.g., mothers’ and fathers’ ethnicity, education, income, and marital status) and stressors (i.e., depression, addictions, adverse childhood experiences) which might affect RF, from surveys completed at clinic visits or sent in by mail. Trained interviewers, the authors LA and MH, conducted the PDI semi-structured interview (Slade et al., 2004) to assess parents’ RF and participants completed the PRFQ (Fonagy et al., 2016) and RFQ (Luyten et al., 2012a).

To determine descriptive statistics of the sample, we assessed depression with the Edinburgh Postnatal Depression Scale (EPDS; Cox et al., 1987; Cox et al., 2014), addictions with the Alcohol, Smoking, and Substance Involvement Screening Test questionnaire (ASSIST; World Health Organization Assist Working Group, 2002) and childhood adversities with the Adverse Childhood Experiences (ACE; Felitti et al., 1998) questionnaire. The EPDS is a 10-item self-report questionnaire that measures depression with specificity of 67.7%, and sensitivity of 66.7–69% (Cox et al., 1987). A cut-off point of ≥ 13 typically indicates greater likelihood of major depressive disorder (Khalifa et al., 2015). The ASSIST questionnaire measures drug addiction and dependence on alcohol, cannabis, cocaine, amphetamine, sedatives or sleeping pills, and other drugs (inhalants, hallucinogens, opioids, etc.) over the lifetime. The ASSIST questionnaire exhibits high internal consistency and validity (construct, concurrent, and discriminant) (Humeniuk and Ali, 2006). ASSIST scores three or less (10 for alcohol) indicate a lower risk of problems associated with the use of the substance involved (World Health Organization Assist Working Group, 2002). The ACE questionnaire is a 10-item self-report measure used to measure the association between multiple types of abuse experienced before age 18 years and various types of health outcomes (Felitti et al., 1998). Murphy et al. (2014) determined excellent internal consistency (α = 0.88) and validity of the ACE Questionnaire.

### Measures of RF

#### The Reflective Function Scale (RFS; Fonagy et al., 1998) Applied to the Parent Development Interview (PDI; Slade et al., 2004)

The PDI is a 20-item semi-structured interview used to examine parents’ representations of their child in the context of the parent–child relationship. Conducted in separate interviews with each parent, the PDI takes approximately 1 h to complete. This narrative was audio-recorded and transcribed for coding by RFS-trained coders (Fonagy et al., 1998). Applying the RFS coding scheme to the PDI is designed to measure a person’s capacity to reflect on his/her own as well as others’ mental states from the narrative depictions of behavior and reflections of self and others in relational or mutual contexts. Passages in the interview are scored on an 11-point scale ranging from negative or antireflective RF (−1 = low RF) to full or exceptional RF (9 = high RF) based on exhibition of criteria such as awareness and identification of one’s own and other’s mental states, the recognition of limitations to reflect on and ability to demonstrate awareness of diverse perspectives (Slade et al., 2004). RF scores are generated in 3 subscales: PDI-rated RFS Self score, PDI-rated RFS Child score and PDI-rated RFS Total score (Slade et al., 2004). Scores of 5 or above indicate higher parental RF (Slade et al., 2005), while scores of 4 or less indicate lower parental RF (Kelly et al., 2005; Levy et al., 2006; Taubner et al., 2013).

Fonagy’s 11-point RFS has acceptable reliability and validity (Bouchard et al., 2008). In our study, author MH coded 90 interviews and a master coder/trainer from The New School University, NYC, United States coded 60 interviews. To assess inter-rater reliability, the PDI-rated RFS scores were grouped into two categories with higher RF > 3 and lower RF ≤ 3, based on the original scale rating of −1 to +9 and based on the average RF score of 3 in our sample. Coder agreement was assessed on 10% (*n* = 15) of PDI’s double coded for RF by author MH and the master RF coder. They achieved > 80% agreement overall, with 86% for PDI-rated RFS Total scores and PDI-rated RFS Self scores and 80% for the PDI-rated RFS Child scores.

#### Reflective Function Questionnaire (RFQ; Fonagy et al., 2016)

The most recent version of the RFQ is an 8-item self-reported questionnaire developed to capture RF in general. It takes 3 min to administer. The questionnaire consists of two subscales including: RFQ Certainty and RFQ Uncertainty about mental states. Responses are rated on a 7-point Likert scale which contains answers ranging from 1 to 7 (*Strongly Agree*). The RFQ Certainty subscale measures the extent to which a person is certain about mental states and is assessed by how much they disagree with statements such as “People’s thoughts are a mystery to me.” The 7-point item is rescored (3, 2, 1, 0, 0, 0, 0 with 3 = disagree strongly) in such a way that agreement to any degree (or a neutral response) demonstrates more optimal mentalizing (awareness of the opaqueness of mental states), and strong disagreement reflects hyper-mentalizing. With the RFQ Uncertainty subscale, uncertainty about mental states is assessed by the degree to which a person agrees with statements such as “Sometimes I do things without really knowing why” and is rescored (0, 0, 0, 0, 1, 2, 3; with 3 = agree strongly). High scores represent hypomentalizing, and lower scores represent more optimal mentalizing (Cucchi et al., 2018; Handeland et al., 2019). Scores range from 0 to 3. The RFQ exhibits good reliability and validity. Currently, the RFQ has no well-established clinical cut-offs for its subscales (Peter Fonagy, personal communication, March 27, 2020). Cronbach alpha coefficients for our sample are reported in Table 4.

#### Parental Reflective Function Questionnaire (PRFQ; Luyten et al., 2017a)

The most recent version of the PRFQ is an 18-item self-reported questionnaire that assesses parental RF and takes 5 min to administer. It includes items related to parental interest and curiosity in their child’s mental states and how these mental states may have an impact on behavior. Luyten et al. (2017b) developed three subscales to capture key features of parental RF including: (a) Pre-mentalizing subscale to assess non-mentalizing modes specific to parents with maladaptation in RF (e.g., inability to dive into the subjective experiences of the child), (b) Certainty in Mental States subscale to examine the capacity to identify and understand the opaqueness of mental states, and (c) Interest and Curiosity subscale that relates to parental interest and curiosity in mental states (Slade, 2005; Slade et al., 2007; Steele et al., 2008). The subscales were identified by exploratory factor and verified by confirmatory factor analysis (Rutherford et al., 2015). Each subscale consists of 6 items and each item is rated on a 7-point Likert scale. Items #11 and #16 are reverse coded and then the mean is calculated for the six items (University College London, 2020). According to the questionnaire developers (Luyten et al., 2017b), items were created based on descriptions and exemplars in the RF manuals for the AAI (Fonagy et al., 1998) and PDI (Slade et al., 2007). As shown, the PRFQ has good internal consistency. Scores from each sub-scale range from 1 to 7. Cronbach’s alpha coefficients for our sample are presented in Table 4.

The PRFQ has no well-established clinical cut-offs for its subscales (Luyten, personal communication, March 27, 2020). For the PRFQ Pre-mentalizing subscale, higher scores indicate lower levels of parental RF (Luyten et al., 2017a; Pazzagli et al., 2018). For the PRFQ Certainty in Mental States and Interest and Curiosity subscales, scoring to predict high or low scores was less clear, with the literature referring to either: (a) high scores indicating high RF, or (b) both low and very high scores indicating lower RF. In other words, average levels of both PRFQ Certainty in Mental States and Interest and Curiosity subscales may be more optimal, whereas either low or very high levels may be more dysfunctional (Luyten et al., 2017b).

#### Scoring Decisions

To aid in interpreting results, we made the decision to ensure that higher scores on all subscales for RFQ and PRFQ measures were consistent with higher RF by reverse scoring as appropriate. For the two variables PRFQ Certainty in Mental States and Interest and Curiosity in which either high scores or middling scores may be most consistent with higher RF (Luyten et al., 2017a), we transformed these variables by squaring the deviations from the sample mean for the two PRFQ subscales [score = (y − mean)^2^] (Mood et al., 1974). This approach created continuous variables for PRFQ Certainty in Mental States and PRFQ Interest and Curiosity scores with the recoded lower scores (closer to original mean) considered more optimal RF and higher scores would be lower RF. Again, to ensure that all interpreted variables were consistent with the interpretation of higher scores indicating higher RF, we reverse scored the transformed variables (see Table 1 for details). However, to be exhaustive, we report on both the (a) untransformed and (b) transformed and reverse scored versions of each variable.

**TABLE 1:** Description of reflective function (RF) measures.

Measure	Scoring	Higher scores	Reverse scored?	After reverse scoring, higher scores
**PDI-rated reflective function scale (RFS)**				
PDI-rated RFS child subscale	−1 to 9	Higher RF (Fonagy et al., 1998)	No	N/A
PDI-rated RFS self subscale	−1 to 9	Higher RF (Fonagy et al., 1998)	No	N/A
PDI-rated RFS total subscale	−1 to 9	Higher RF (Fonagy et al., 1998)	No	N/A
**Reflective function questionnaire (RFQ)**				
RFQ certainty subscale	0–3	Higher RF (Cucchi et al., 2018)	No	N/A
RFQ uncertainty subscale	0–3	Lower RF (Cucchi et al., 2018)	Yes	Higher RF
**Parental reflective function questionnaire (PRFQ)**				
PRFQ pre-mentalizing subscale	1–7	Lower RF (Luyten et al., 2017a)	Yes	Higher RF
PRFQ interest and curiosity subscale	1–7	Higher RF (Luyten et al., 2017a)	No	N/A
PRFQ interest and curiosity subscale-transformed	0–36^a^	Lower RF (Luyten et al., 2017a)	Yes	Higher RF
PRFQ certainty in mental states subscale	1–7	Higher RF (Luyten et al., 2017a)	No	N/A
PRFQ certainty in mental states subscale-transformed	0–36^a^	Lower RF (Luyten et al., 2017b)	Yes	Higher RF

**^*a*^Individual deviations from the study sample mean includes a possible 0 for no difference from sample mean or an individual difference of 6 (if mean = 1 or 7). Squaring these deviations gives a possible maximum value of 36. [Transformed variable = (y − mean)^2^, range = 0–36].**

### Data Analysis

We analyzed data using IBM SPSS Statistics 26.0. We analyzed demographic and descriptive features of the sample with assessment of central tendency and frequencies. To ensure mothers’ and fathers’ scores were different from each other or did not differ based on membership in a given couple/family (that is, a given parent’s score was not influenced by their couple/family or not “independent”), prior to grouping for analyses, we conducted intraclass correlations, correlations, and examined scatterplots. Mothers’ and fathers’ scores were not correlated and were thus deemed independent. Nonetheless, we included a variable called “gender” to enable examination of mothers’ and fathers’ scores. We also investigated the descriptive properties of RF measures as shown in Table 3.

Then, we conducted reliability analysis to determine the internal consistency of the items of the questionnaires (PRFQ and RFQ) measured by using Cronbach’s alpha coefficients (Tavakol and Dennick, 2011). In order to study the relationship between RF scores from PDI-rated RFS and the questionnaires (PRFQ and RFQ), we computed Pearson’s correlation (Cohen, 1988; Benesty et al., 2009). We then regressed the significant RF questionnaire subscales scores on to PDI-rated RFS scores in separate multiple regression analyses (Iwasaki, 2020) with parents’ gender as a covariate.

We examined Cohen (1960) kappa, a measure of agreement (Harris and Brown, 2010) among the subscale scores and PDI-rated RFS scores using typical, exploratory, clinically meaningful quartiles at 25th (low) and 75th (high) percentile cut-offs. This also enabled the observation of associations by comparing extremes in this community sample. Reverse scoring was employed, as mentioned above, to enable interpretation of higher scores consistent with higher RF. However, to ensure transparency, we also report results from untransformed variables as well as results from variables with extreme outliers removed. We performed tests only with the significant subscales (from RFQ or PRFQ with PDI-rated RFS scores) identified in earlier analyses.

Specifically, we then determined the accuracy, that is, we examined the low and high categories for the gold standard PDI-rated RFS, to assess agreement with low and high categories on the PRFQ measure. We determined the sensitivity, that is, whether the PRFQ correctly identified low scores on the gold standard. We examined specificity, that is, whether the PRFQ correctly identified high scores on the gold standard. We also calculated the positive predictive value (i.e., among those detected by the PRFQ as low RF, how many are actually low as measured by the gold standard) and negative (i.e., among those detected by the PRFQ as high, how many are actually high as measured by the gold standard) (Yusoff, 2010). For all analyses, alpha was set at 0.05, two-tailed.

## Results

The characteristics of our sample are depicted in Table 2. Mothers (*n* = 75) and fathers (*n* = 75) completed the PDI interviews and the questionnaire measures of PRFQ and RFQ with no missing data. Table 3 presents the descriptive statistics of the RF measures (PDI-rated RFS, RFQ, and PRFQ), with the mean PDI-rated RFS scores ranging from 3.13 to 3.18 on the scale of −1 to 9. Table 4 shows that the Cronbach’s alpha ranged from 0.69 to 0.78 for the PRFQ and RFQ which demonstrates satisfactory internal consistency (Tavakol and Dennick, 2011). To determine convergent validity, Tables 5, 6 present the results of Pearson correlation analysis and subsequent multiple linear regression models, respectively, for the correlated variables. Of the three subscales of the PRFQ, we found significant correlations among the PRFQ Interest and Curiosity subscale (untransformed variable) and the PRFQ Certainty in Mental States (transformed and reverse—scored variable) with the PDI-rated Self, Child, and Total scores. We also found a significant correlation between PRFQ Pre-mentalizing and PDI-rated RFS Child scores, with trends approaching significance for PDI-rated RFS Self scores.

**TABLE 2:** Sociodemographic profile of the sample (*n* = 150).

Sociodemographic profile	Mothers (*n* = 75)		Fathers (*n* = 75)		Total (*n* = 150)	
	*n*	%	*n*	%	*n*	%
Number of participants	75	100%	75	100%	150	100%
**Ethnicity**						
Caucasian	64	85%	63	84%	127	85%
Non-Caucasian	11	15%	12	16%	23	15%
**First language**						
English	59	79%	65	87%	124	83%
Not English	16	21%	10	13%	26	17%
**Marital status**						
Married	72	96%	72	96%	144	96%
Common-law	3	4%	3	4%	6	4%
**Education**						
University	60	80%	48	64%	108	72%
College/technical	12	16%	22	29%	34	23%
High school	3	4%	5	7%	8	5%
**Employment**						
Employed	51	68%	71	95%	122	81%
Unemployed	24	32%	4	5%	28	19%
**Number of children in household (Mean ± SD)**						
	2.25 ± 0.76		2.28 ± 0.76		2.27 ± 0.76	
**Edinburgh postnatal depression scale (EPDS) scores (*Mean ± SD)***						
	4.29 ± 3.36		6.16 ± 4.41		5.34 ± 4.12	
**Alcohol, smoking, and substance involvement screening (ASSIST) Scores (*Mean ± SD)***						
Total alcohol score	5.73 ± 3.46		7.85 ± 5.82		6.79 ± 4.89	
Total Cannabis score	1.12 ± 2.09		1.94 ± 4.25		1.53 ± 3.37	
Total Cocaine score	0.09 ± 0.40		0.14 ± 0.53		0.12 ± 0.47	
Total amphetamine score	0.13 ± 0.34		0.14 ± 0.35		0.14 ± 0.35	
Total sedatives or sleeping pills	0.11 ± 0.42		0.18 ± 0.63		0.15 ± 0.54	
Total other drugs	0.21 ± 0.66		0.28 ± 0.78		0.25 ± 0.72	
**Adverse childhood experiences (*Mean ± SD)***						
	1.24 ± 1.66		0.84 ± 0.19		1.05 ± 0.17	

**TABLE 3:** Descriptive statistics of RF measures.

RF measures	*N*	Mean	SD	Min	Max	Percentiles		
						25th	50th	75th
**Reflective function scale (RFS)**								
PDI-rated self RFS scores	150	3.18	1.49	−1.00	7.00	2.00	3.00	4.00
PDI-rated child RFS scores	150	3.13	1.40	0.00	7.00	2.00	3.00	4.00
PDI-rate total RFS scores	150	3.18	1.39	0.00	7.00	2.00	3.00	4.00
**Parental reflective function questionnaire (PRFQ)**								
PRFQ pre-mentalizing scores	150	1.86	0.75	1.00	5.17	1.33	1.67	2.17
PRFQ pre-mentalizing scores-*reverse coded*^*a*^	150	5.14	0.75	1.83	6.00	4.83	5.33	5.67
PRFQ certainty in mental states scores	150	3.56	1.04	1.50	6.17	2.67	3.58	4.38
PRFQ certainty in mental states scores-*transformed*^*b*^	150	1.08	1.15	0.00	6.84	0.28	0.78	1.50
PRFQ certainty in mental states scores-*transformed and reverse-coded*^*c*^	150	5.92	1.15	0.18	7.00	5.51	6.21	6.72
PRFQ interest and curiosity scores	150	5.96	0.71	2.00	7.00	5.50	6.00	6.50
PRFQ interest and curiosity scores-*transformed*^*b,d*^	149	0.40	0.51	0.00	3.95	0.03	0.24	0.67
PRFQ interest and curiosity scores-*transformed and reverse-coded*^*c,d*^	149	6.60	0.51	3.05	7.00	6.33	6.76	6.97
**Reflective function questionnaire (RFQ)**								
RFQ certainty score	150	1.20	0.74	0.00	3.00	0.67	1.17	1.67
RFQ uncertainty score	150	0.42	0.47	0.00	1.83	0.00	0.33	0.67
RFQ uncertainty score-*reverse coded*^*a*^	150	6.58	0.47	5.17	7.00	6.33	6.67	7.00

**^*a*^Reverse coded so that higher scores are consistent with higher RF. ^*b*^Transformed by taking the square of the deviation from the mean: (y − mean)^2^. ^*c*^Transformed by taking the square of the deviation from the mean: (y − mean)^2^ and then reverse scored so that higher scores are consistent with higher RF. ^*d*^Extreme outlier was removed to stabilize the mean.**

**TABLE 4:** Descriptive statistics of the outcome variables (questionnaire domains) and Cronbach’s alpha (as per original untransformed coding).

Subscale	Subscale questions	*n*	Mean	SD	Cronbach’s alpha
PRFQ pre-mentalizing		150	1.86	0.753	0.688
	PRFQ_Q_1	150	1.50	1.022	
	PRFQ_Q_4	150	1.44	1.096	
	PRFQ_Q_7	150	3.01	1.751	
	PRFQ_Q_10	150	1.39	0.865	
	PRFQ_Q_13	150	1.69	0.991	
	PRFQ_Q_16	150	2.15	1.289	
PRFQ certainty in mental states		150	3.56	1.043	0.772
	PRFQ_Q_2	150	3.60	1.461	
	PRFQ_Q_5	150	2.85	1.632	
	PRFQ_Q_8	150	3.37	1.509	
	PRFQ_Q_11R	150	3.19	1.450	
	PRFQ_Q_14	150	4.49	1.553	
	PRFQ_Q_17	150	3.83	1.549	
PRFQ interest and curiosity		150	5.96	0.710	0.745
	PRFQ_Q_3	150	6.11	1.024	
	PRFQ_Q_6	150	5.41	1.342	
	PRFQ_Q_9	150	6.32	0.838	
	PRFQ_Q_12	150	5.99	0.934	
	PRFQ_Q_15	150	5.99	0.959	
	PRFQ_Q_18R	150	5.95	1.241	
RFQ certainty		150	1.20	0.740	0.784
	RFQ_Qc_1	150	0.90	0.947	
	RFQ_Qc_2	150	1.39	1.111	
	RFQ_Qc_3	150	1.37	1.096	
	RFQ_Qc_4	150	0.95	1.086	
	RFQ_Qc_5	150	1.13	1.151	
	RFQ_Qc_6	150	1.47	1.008	
RFQ uncertainty		150	0.42	0.468	0.761
	RFQ_Qu_2	150	0.39	0.694	
	RFQ_Qu_4	150	0.59	0.779	
	RFQ_Qu_5	150	0.45	0.756	
	RFQ_Qu_6	150	0.24	0.539	
	RFQ_Qu_7	150	0.39	0.675	
	RFQ_Qu_8	150	0.45	0.691	

**TABLE 5:** Pearson’s product moment correlation coefficients.

Questionnaire subscales	PDI-rated RFS self scores		PDI-rated RFS child scores		PDI-rated RFS total scores	
	Pearson’s r	*p*-value	Pearson’s r	*p*-value	Pearson’s r	*p*-value
PRFQ pre-mentalizing scores	−0.146	0.075	−**0.180**	**0.028**	−0.155	0.059
PRFQ pre-mentalizing scores-*reverse coded*^a^	0.146	0.075	0.180	0.028	0.155	0.059
PRFQ certainty in mental states scores	−0.084	0.309	−0.081	0.323	−0.117	0.153
PRFQ certainty in mental states scores-*transformed and reverse coded*^b^	**0.218**	**0.007**	**0.217**	**0.008**	**0.236**	**0.004**
PRFQ interest and curiosity scores	**0.224**	**0.006**	**0.298**	**< 0.001**	**0.271**	**0.001**
PRFQ interest and curiosity scores-*transformed and reverse coded*^b^	−0.146	0.076	−0.140	0.089	−0.157	0.056
RFQ certainty scores	0.018	0.827	0.006	0.944	−0.032	0.699
RFQ uncertainty scores	−0.043	0.601	−0.002	0.983	0.026	0.751
RFQ uncertainty scores-*reverse coded*^a^	0.043	0.601	0.002	0.983	−0.026	0.751

**^*a*^Reverse coded so that higher scores are consistent with higher RF. ^*b*^Transformed by taking the square of the deviation from the mean: (y − mean)^2^ and then reverse scored so that higher scores are consistent with higher RF. PRFQ certainty in mental states scores-transformed and reverse coded scores were significantly correlated with PDI-rated RFS Self, Child and Total Scores. PRFQ interest and curiosity scores (without transformations) were significantly correlated with PDI-rated RFS Self, Child, and Total Scores.**

**TABLE 6:** Linear regression coefficients for outcome variables correlated with PDI-rated RFS scores.

Questionnaire subscale (dependent)	Predictors (independent)	Regression coefficients		*t*	*p*-value
		B	SE (B)		
PRFQ pre-mentalizing^a^ (Y1)	(Constant)	4.829	0.157	30.825	< 0.001
	Parent gender	0.016	0.122	0.127	0.899
	PDI-rated RFS child scores	0.096	0.044	2.190	0.030
PRFQ certainty in mental states^b^ (Y2)	(Constant)	5.348	0.227	23.561	< 0.001
	Parent gender	0.109	0.186	0.583	0.561
	PDI-rated RFS self scores	0.162	0.063	2.595	0.010
PRFQ certainty in mental states^b^ (Y2)	(Constant)	5.312	0.237	22.395	< 0.001
	Parent gender	0.126	0.185	0.678	0.499
	PDI-rated RFS child scores	0.174	0.066	2.616	0.010
PRFQ certainty in mental states^b^ (Y2)	(Constant)	5.265	0.238	22.130	< 0.001
	Parent gender	0.103	0.186	0.554	0.580
	PDI-rated RFS Total scores	0.189	0.067	2.836	0.005
PRFQ interest and curiosity^c^ (Y3)	(Constant)	5.556	0.139	40.059	< 0.001
	Parent gender	0.190	0.114	1.665	0.098
	PDI-rated RFS self scores	0.097	0.038	2.541	0.012
PRFQ interest and curiosity^b,d^ (Y3)	(Constant)	5.415	0.142	38.151	< 0.001
	Parent gender	0.188	0.111	1.693	0.093
	PDI-rated RFS child score	0.144	0.040	3.618	< 0.001
PRFQ interest and curiosity^b,d^ (Y3)	(Constant)	5.460	0.144	37.844	< 0.001
	Parent gender	0.180	0.113	1.599	0.112
	PDI-rated RFS total score	0.129	0.041	3.182	0.002

**^*a*^Reverse coded. ^*b*^Transformed by taking the square of the deviation from the mean: (y − mean)^2^ and then reverse scored. ^*c*^Original untransformed variable. ^*d*^Extreme outlier was removed to stabilize the mean.**

To elaborate, scores on the PRFQ Certainty in Mental States (transformed and reverse scored) and PRFQ Interest and Curiosity (original scoring) subscales correlated significantly with the gold standard PDI-rated RFS, Fonagy et al. (1998) 11-point scale. In other words, higher scores correlated with higher RF for those sub-scales. Also, scores on the PRFQ Pre-mentalizing (original scoring) correlated negatively with the PDI-rated RFS Child scores in that higher pre-mentalizing was indicative of lower RF toward the Child. Thus, our hypothesis was supported. Linear regression models confirmed these relationships, accounting for parent gender. None of the subscales of RFQ were significantly correlated with PDI-rated RFS Total scores.

Cohen’s kappa coefficients of agreement among the PRFQ and RFQ subscale scores and PDI-rated RFS scores are presented in Table 7. Of the significant kappa statistics (all but PRFQ Pre-mentalizing), weak agreements were noted ranging from 0.16 for PRFQ Certainty in Mental States to 0.25 for Interest and Curiosity with higher scores indicated higher RF. Finally, the results of sensitivity and specificity of the PRFQ subscales with positive and negative predictive values to detect “Low” (< 2, i.e., 25th percentile) PDI-rated RFS scores are presented in Table 8. Positive predictive values ranged from 37 to 45%, showing that PRFQ subscale scores had low predictive ability to detect low RF on the PDI-rated RFS subscales. In contrast, negative predictive values ranged from 76 to 80% showing that the PRFQ subscale scores had high predictive ability to detect high RF on the PDI-rated RFS subscales.

**TABLE 7:** Measure of agreement among the questionnaire subscale scores correlated with PDI rated scores using the 25th percentile values as cut-off points for low and high scores.

		Low	High	Agreement	Kappa	*p*-value
**PDI-rated RFS child scores (*n* = 150)**						
PRFQ pre-mentalizing scores^a^	Low	15	26	96	0.107	0.188
		10.0%	17.3%			
	High	28	81	64.0%		
		18.7%	54.0%			
**PDI-rated RFS self scores (*n* = 150)**						
PRFQ certainty in mental state scores^b^	Low	18	27	99	0.175	0.032
		12.0%	18.0%			
	High	24	81	66.0%		
		16.0%	54.0%			
**PDI-rated RFS child scores (*n* = 150)**						
PRFQ certainty in mental state scores^b^	Low	18	27	98	0.164	0.044
		12.0%	18.0%			
	High	25	80	65.3%		
		16.7%	53.3%			
**PDI-rated RFS total scores (*n* = 150)**						
PRFQ certainty in mental state scores^b^	Low	17	28	100	0.175	0.031
		11.3%	18.7%			
	High	22	83	66.7%		
		14.7%	55.3%			
**PDI-rated RFS self scores (*n* = 149)**						
PRFQ interest and curiosity scores^c^	Low	15	25	98	0.135	0.098
		10.1%	16.8%			
	High	26	83	65.8%		
		17.4%	55.7%			
**PDI-rated RFS child scores (*n* = 149)**						
PRFQ interest and curiosity scores^c^	Low	14	26	95	0.092	0.263
		9.4%	17.4%			
	High	28	81	63.8%		
		18.8%	54.4%			
**PDI-rated RFS total scores (*n* = 149)**						
PRFQ interest and curiosity scores^c^	Low	13	27	97	0.097	0.235
		8.7%	18.1%			
	High	25	84	65.1%		
		16.8%	56.4%			
**PDI-rated RFS self scores (*n* = 150)**						
PRFQ interest and curiosity scores^d^	Low	18	28	98	0.165	0.043
		12.0%	18.7%			
	High	24	80	65.3%		
		16.0%	53.3%			
**PDI-rated RFS child scores (*n* = 150)**						
PRFQ interest and curiosity scores^d^	Low	21	25	103	0.250	0.002
		14.0%	16.7%			
	High	22	82	68.7%		
		14.7%	54.7%			
**PDI-rated RFS total scores (*n* = 150)**						
PRFQ interest and curiosity scores^d^	Low	18	28	101	0.198	0.015
		12.0%	18.7%			
	High	21	83	67.3%		
		14.0%	55.3%			

**^*a*^Reverse coded. ^*b*^Transformed by taking the square of the deviation from the mean: (y − mean)^2^ and then reverse scored. ^*c*^Extreme outlier was removed to stabilize the mean followed by transformation by taking the square of the deviation from the mean: (y − mean)^2^ and then reverse scoring. ^*d*^Original untransformed variable.**

**TABLE 8:** Sensitivity and specificity of the questionnaire subscales to detect “Low” and “High” PDI-rated RFS scores using the 25th percentile values as cut-off points for low and high scores.

		Low	High	Accuracy	Sensitivity	Specificity	PPV	NPV
**PDI-rated RFS child scores (*n* = 150)**								
PRFQ pre-mentalizing scores^*a*^	Low	15	26	0.640	0.349	0.757	0.366	0.743
		10.0%	17.3%					
	High	28	81					
		18.7%	54.0%					
**PDI-rated RFS self scores (*n* = 150)**								
PRFQ certainty in mental state scores^*b*^	Low	18	27	0.660	0.429	0.750	0.400	0.771
		12.0%	18.0%					
	High	24	81					
		16.0%	54.0%					
**PDI-rated RFS child scores (*n* = 150)**								
PRFQ certainty in mental state scores^*b*^	Low	18	27	0.653	0.419	0.748	0.400	0.762
		12.0%	18.0%					
	High	25	80					
		16.7%	53.3%					
**PDI-rated RFS total scores (*n* = 150)**								
PRFQ certainty in mental state scores^*b*^	Low	17	28	0.667	0.436	0.748	0.378	0.790
		11.3%	18.7%					
	High	22	83					
		14.7%	55.3%					
**PDI-rated RFS self scores (*n* = 149)**								
PRFQ interest and curiosity scores^*c*^	Low	15	25	0.658	0.366	0.769	0.375	0.761
		10.1%	16.8%					
	High	26	83					
		17.4%	55.7%					
**PDI-rated RFS child scores (*n* = 149)**								
PRFQ interest and curiosity scores^*c*^	Low	14	26	0.638	0.333	0.757	0.350	0.743
		9.4%	17.4%					
	High	28	81					
		18.8%	54.4%					
**PDI-rated RFS total scores (*n* = 149)**								
PRFQ interest and curiosity scores^*c*^	Low	13	27	0.651	0.342	0.757	0.325	0.771
		8.7%	18.1%					
	High	25	84					
		16.8%	56.4%					
**PDI-rated RFS self scores (*n* = 150)**								
PRFQ interest and curiosity scores^*d*^	Low	18	28	0.653	0.429	0.741	0.391	0.769
		12.0%	18.7%					
	High	24	80					
		16.0%	53.3%					
**PDI-rated RFS child scores (*n* = 150)**								
PRFQ interest and curiosity scores^*d*^	Low	21	25	0.687	0.488	0.766	0.457	0.788
		14.0%	16.7%					
	High	22	82					
		14.7%	54.7%					
**PDI-rated RFS total scores (*n* = 150)**								
PRFQ interest and curiosity scores^*d*^	Low	18	28	0.673	0.462	0.748	0.391	0.798
		12.0%	18.7%					
	High	21	83					
		14.0%	55.3%					

**PPV, positive predictive values; NPV, negative predictive values. ^*a*^Reverse coded. ^*b*^Transformed by taking the square of the deviation from the mean: (y − mean)^2^ and then reverse scored. ^*c*^Extreme outlier was removed to stabilize the mean followed by transformation by taking the square of the deviation from the mean: (y − mean)^2^ and then reverse scoring. ^*d*^Original untransformed variable.**

## Discussion

Our data provided preliminary evidence for the convergent validity of the PRFQ to the PDI-rated RFS scores, along with acceptable internal consistency reliability of its subscales. None of the subscales of the RFQ correlated with the PDI-rated RFS scores. Analyses employed the newly validated versions of the PRFQ (Luyten et al., 2017a) and RFQ (Fonagy et al., 2016), hence complementing to existing research (Cucchi et al., 2018) with more robust measures. Given the sample means demonstrating low risk for depression on the EPDS, low risk for substance abuse on the ASSIST, and low early adversity on the ACE questionnaire, these findings may be applicable to other community (non-clinical) samples.

The findings from our study are supported by existing literature (Slade et al., 2005, 2007). High levels of parental RF are thought to be explicated in keen interest and curiosity in mental states that leads to an exploration for understanding (Slade et al., 2004, 2007). High ratings on the PDI-rated RFS indicate an increasingly explicit and refined understanding of how mental states work and impact behavior (Slade et al., 2004). Accordingly, parents’ higher levels of interest and curiosity in reflecting about their child’s subjective experience and taking their own mental states and their child’s perspective were correlated. Overall, a parent’s interest and curiosity in mental states reflects certainty in her/his knowledge about her/his child, resulting in greater communication and involvement with the child.

In contrast, lower PRFQ Pre-mentalizing scores, demonstrating lower capacity for demonstrating a genuine curiosity in the subjective experience of the child, were correlated with lower PDI-rated RFS Child scores. Our findings demonstrated the degree to which the participants reported struggling in comprehending their children’s mental states accurately which is supported by previous research (Slade et al., 2007; Luyten et al., 2017b). The relationship between the PRFQ Pre-mentalizing and the PDI-rated RFS scores agrees with existing research conducted on the PRFQ (Rostad and Whitaker, 2016; Luyten et al., 2017a). Accordingly, if a parent finds it challenging to understand the child’s internal world, he or she may find it challenging to think or talk about the child’s mental states. This aligns with suggestions put forward by the RF researchers that these pre-mentalizing modes of understanding mental states from parents’ perspective are often associated with making malicious attributions and an inability to understand and interpret child’s internal subjective world (Rostad and Whitaker, 2016), the features that research suggests are characteristic of parents with severe mentalizing problems (Slade et al., 2005; Suchman et al., 2010).

Interestingly, the parents’ scores on PRFQ Certainty in Mental States (transformed and reverse coded) correlated positively with PDI-rated RFS scores, while the untransformed variable did not. This finding is supported by existing research (Luyten et al., 2017b) demonstrating that average levels of PRFQ Certainty in Mental States and Interest and may be most optimal, whereas either low or very high levels of RF may be more malevolent. Theoretically, the development of pathological levels of RF demonstrates that being highly certain of others’ mental states (e.g., thoughts, feelings) tend to be a type of RF deficit (Fonagy et al., 2003). One persuasive explanation may be that the parents, who have high levels of certainty about mental states, may attain a PRFQ Certainty in Mental States score that, in fact, does not reflect their real RF capacity, because their pseudo-mentalizing statements may come from statements that are given high RF scores (Handeland et al., 2019). Stated simply, too low or too high levels of certainty about mental states can lead to rigidity and a collapse of RF (Fonagy et al., 2016).

Also, we did not find significant associations between the PDI-rated RFS scores and the RFQ, which implies that the RFQ may not be useful to capture the essence of the parental RF. While we expected this, as the PDI assesses parenting in the current context and not family of origin context, as assessed with the AAI, our findings contrast with others. Handeland et al. (2019) found that the RFQ Uncertainty was significantly negatively associated with PDI-rated RFS Self scores. However, Handeland et al. (2019) used the Norwegian version of the RFQ-8 which is relatively new and has no well-established or validated cut-off for clinically high scores on its scales. The cut-offs in the study were based on an assumption that a mean score of at least 1 on either of these scales represents a marked RF deficit. Also, the findings from their study were not supported by the fact that the RFQ Certainly was not correlated with PDI-rated RFS Self scores. Given the strong negative correlation between the RFQ Certainty and Uncertainty scales (Fonagy et al., 2016; Cucchi et al., 2018; Handeland et al., 2019), to a certain extent, findings demonstrating low scores on one scale need to be coupled with high scores on the other, and vice versa.

To our knowledge, our study stands to be the first to compare the PRFQ with the RFS from the PDI. As suggested, the time- and cost-intensiveness of the RFS applied to an interview, is addressed by the quick and efficient PRFQ. Reducing the patient/participant burden of assessing RF will improve feasibility of administration in clinical and research settings. Further, while the RFS applied to the PDI generally yields global scores of participants’ RF, the PRFQ yields information about specific dimensions of mentalizing—pre-mentalizing and certainty, interest, and curiosity about mental states, which may have important implications for clinicians and researchers (Fonagy et al., 2016). As a result, the PRFQ, as a reliable and valid clinically meaningful self-report measure of RF, may be best suited for administration in wide-ranging settings.

The current research has several strengths including an adequate sample size of both mothers and fathers and “master” level coding of PDI interviews for RF. Moreover, while the PRFQ has been validated mostly in the small, clinical or at-risk samples to date (Luyten et al., 2017b), we report the validity of the PRFQ in a large normative community sample. We conclude that all three subscales of the PRFQ have satisfactory convergent validity with the PDI-rated RFS. Given that the PRFQ scores show high specificity (negative predictive values ranging from 76 to 80%) in predicting high RF scores, the PRFQ may replace the PDI-rated RFS when scores are high on the PRFQ, as may be expected in low-risk samples. However, PRFQ scores exhibit low sensitivity scores (positive predictive values ranging from 37 to 45%) in predicting low RF scores, thus for high-risk samples, it may be recommended to follow up with the PDI interview when the RF scores are low on the PRFQ. A potential limitation is that participants may attempt to appear more reflective than they are in an attempt to please the researcher, as in the case of acquiescent response or social desirability. As this will be an ongoing limitation of any test of RF (De Roo et al., 2019), caution should be taken before forensic use, in for example, court cases related to child custody. This is a problem with any self-report measure. Given that this is the first study of its kind, a replication of the current project that includes Adult Attachment Interview (AAI; George et al., 1996) in the mix with the RFS is recommended for future research.

## Conclusion

Our evaluation of convergent validity demonstrated that the PRFQ is significantly correlated with the gold standard RFS applied to the PDI in measuring theoretically similar concepts of RF in parents. The results from our study provide evidence for the reliability and validity of the PRFQ as brief multifaceted measure of parental RF, offering potentially valuable methodological approaches for studies of parental development, especially in low-risk populations.

## Data Availability Statement

The data analyzed in this study is subject to the following licenses/restrictions: N/A. Requests to access these datasets should be directed to LA, lanis@ucalgary.ca.

## Ethics Statement

The studies involving human participants were reviewed and approved by Conjoint Health Research Ethics Board, University of Calgary. The patients/participants provided their written informed consent to participate in this study.

## Author Contributions

LA formulated the research question and oversaw all aspects of manuscript preparation from literature review, the data collection, the data analysis, interpreted the results, and undertook writing and submission. GP helped with the data analysis, overseeing the literature review, interpretation of the results, and writing. NL helped formulate the research question, oversaw the literature review and data analysis, and interpreted the results and writing. KB and CE helped oversee the literature review and data analysis, and interpreted the results and writing. MH helped review the literature review, interpretation of the results, and writing. All authors contributed to the article and approved the submitted version.

## Conflict of Interest

The authors declare that the research was conducted in the absence of any commercial or financial relationships that could be construed as a potential conflict of interest.
